# Exciton-dominated Dielectric Function of Atomically Thin MoS_2_ Films

**DOI:** 10.1038/srep16996

**Published:** 2015-11-24

**Authors:** Yiling Yu, Yifei Yu, Yongqing Cai, Wei Li, Alper Gurarslan, Hartwin Peelaers, David E. Aspnes, Chris G. Van de Walle, Nhan V. Nguyen, Yong-Wei Zhang, Linyou Cao

**Affiliations:** 1 Department of Materials Science and Engineering, North Carolina State University, Raleigh, NC 27695, USA; 2 Department of Physics, North Carolina State University, Raleigh, NC 27695, USA; 3 Institute of High Performance Computing, A*STAR, Singapore 138632; 4 Semiconductor and Dimensional Metrology Division, National Institute of Standards and Technology, Gaithersburg, Maryland 20899, USA; 5 Department of Fiber and Polymer Science, North Carolina State University, Raleigh, NC 27695, USA; 6 Materials Department, University of California, Santa Barbara, CA 93106, USA

## Abstract

We systematically measure the dielectric function of atomically thin MoS_2_ films with different layer numbers and demonstrate that excitonic effects play a dominant role in the dielectric function when the films are less than 5–7 layers thick. The dielectric function shows an anomalous dependence on the layer number. It decreases with the layer number increasing when the films are less than 5–7 layers thick but turns to increase with the layer number for thicker films. We show that this is because the excitonic effect is very strong in the thin MoS_2_ films and its contribution to the dielectric function may dominate over the contribution of the band structure. We also extract the value of layer-dependent exciton binding energy and Bohr radius in the films by fitting the experimental results with an intuitive model. The dominance of excitonic effects is in stark contrast with what reported at conventional materials whose dielectric functions are usually dictated by band structures. The knowledge of the dielectric function may enable capabilities to engineer the light-matter interactions of atomically thin MoS_2_ films for the development of novel photonic devices, such as metamaterials, waveguides, light absorbers, and light emitters.

Two-dimensional (2D) transition metal dichalcogenide (TMDC) materials have been known exhibiting strong exciton binding energy that may be one order of magnitude larger than conventional semiconductor materials^1^,^2^,^3^,^4^,^5^,^6^. However, how the extraordinarily strong exciton binding energy could affect the light-matter interactions such as dielectric functions of the materials has remained unexplored. The lack of knowledge about the dielectric function has significantly limited the application of 2D TMDC materials in many exciting photonic fields such as metamaterials^7^, which relies on the sophisticated manipulation of effective dielectric functions to enable novel optical functionalities. In this work we have measured the dielectric function of atomically thin MoS_2_ films and discovered that it is dominated by the effect of the tightly bound excitons, as evidenced by an anomalous dependence of the dielectric function on the layer number. The dielectric function decreases with the layer number increasing when the MoS_2_ films are less than 5 layers thick, but turn to increase with the layer number for thicker. We also quantitatively evaluate the exciton binding energy and Bohr radius of the thin films by fitting the experimental results with an intuitive model. The observed dominance of excitonic effects in the dielectric function is in stark contrast with what expected at conventional materials, whose dielectric functions are usually dictated by band structures^8^,^9^. Our success in this discovery is built upon a unique self-limiting chemical vapor deposition (CVD) process that we have recently developed^10^. The self-limiting CVD process can be used to grow centimeter-scale, uniform, and high quality atomically thin MoS_2_ films with controlled layer numbers and remarkable uniformity (Figs S1–S5). This allows us to examine the dielectric function of MoS_2_ films as a function of well-defined layer numbers. Our work is different from earlier research for the dielectric function of MoS_2_ films^11^,^12^, whose results are likely inaccurate due to the lack of satisfactory uniformity or precise control of the layer number.

We measured the dielectric function (*ε*_1_ + *iε*_2_) of as-grown MoS_2_ films on sapphire substrates using spectroscopic ellipsometry^13^. Figure 1a,b shows the real *ε*_1_ and imaginary *ε*_2_ parts of the dielectric function in the visible range that are derived from experimental measurements (see Figs S7,S8 for the fit between experimental and simulated results). Owing to the extreme geometrical anisotropy of the film, what we obtained is actually the in-plane component of the dielectric tensor because the out-of-plane dielectric function may only contribute trivially to the optical response due to the difficulty in exciting the vertical dipole of the atomically thin film^13^. As further evidence for the measured in-plane dielectric function, we performed the spectroscopic ellipsometry at different incident angles (40°–75°), and all of them ended up with giving very similar dielectric functions. The dielectric function of bulk MoS_2_ is also measured and plotted in Fig. 1a,b as a reference, the result of which is consistent with what reported previously^14^. The three peaks in the spectral dielectric function can be assigned to *A*, *B*, and *C* from low to high energies, respectively^15^,^16^,^17^,^18^. The *A* and *B* peaks are related with the transition from the spin-orbit split valence bands to the lowest conduction band at the *K* and *K’* points, while the *C* peak is associated with the transition from the valence band to the conduction band at the part of the Brillouin zone between the *Λ* and *Γ* point^16^,^17^.

The measured dielectric function is not sensitive to the synthetic process or the substrate. The dielectric functions measured from the MoS_2_ grown by using MoCl_5_ and S as the precursors^10^ and by using MoO_3_ and S as the precursors^19^ are essentially identical (Fig. S9a). We also find that the dielectric functions of the as-grown MoS_2_ films on sapphire substrates and those transferred onto SiO_2_/Si substrates are identical (Fig. S9b). Additionally, the dielectric function of the film is stable under ambient environment. We monitored the dielectric function of the as-grown MoS_2_ films on sapphire substrates as a function of the time for the films to be exposed to ambient environment. We monitored the dielectric function of the films exposed to ambient environment for more than one week and found no change in the measured result (Fig. S10). The result we measured for the monolayer MoS_2_ film is consistent with what previously measured using spectroscopic ellipsometry^20^ but is 10–15% less than the results derived from absorption spectra^21^. We do like to point out some difference in the spectroscopic ellipsometry used by us as well as ref. 20 and the spectroscopic absorption technique used in ref. 21. Spectroscopic ellipsometry is the most established technique for the measurement of dielectric functions, in which two parameters are measured at each wavelength and the dielectric function can be uniquely determined in any spectrum range with the thickness information of the film indepedently determined by AFM. The dielectric function may be derived from spectroscopic reflection using the Kramer-Kronig relationship as well. But to precisely find out the dielectric function using the Kramer-Kronig relationship requires information of the absorption in the entire spectral range. In ref. 21 the absorption of the monolayer in the range higher than 3 eV, whose value is not experimentally available, is assumed to be equal to that of the bulk counterpart. This assumption might overestimate the dielectric function to some degree. We believe this is likely the reason why our result is around 10–15% less than the result reported in ref. 21.

Significantly, the measured dielectric function shows an anomalous dependence on the layer number (Fig. 1a,b). It decreases with the layer number increasing when the film is less than 5–7 layers thick and then turns to increase with the layer for thicker films. We were very careful to ensure no artifact introduced in the measurement. More specifically, we performed extensive AFM for each of the films studied prior to the ellipsometry measurement and confirmed the atomic-scale smoothness (roughness usually < 0.5 nm except the 8L, 9L and 10L films, whose roughness is a little bit larger in the range of 0.7–0.9 nm, see Figs S1–S5) and excellent uniformity of the film. Additionally, for the result of each layer number, we measured at least three different sets of samples and observed only minor variation (5%) in the resulting dielectric function. To further illustrate this anomalous layer-dependence, we extract the imaginary part of the dielectric functions at the *A*, *B*, and *C* peaks from Fig. 1b and plot it as a function of the layer number (Fig. 1c). The result clearly shows a decrease and then an increase in the dielectric function with the layer number continuously increasing from one. The layer dependence is similar for all the *A*, *B*, and *C* peaks (Fig. 1d). Given the similarity in the layer dependence, we only focus on the *C* peak in the following discussion.

To obtain physical insights into the observed layer dependence, we examine the dielectric function from the perspective of quantum mechanics. We only focus on the imaginary part *ε*_2_ because the real part *ε*_1_ can be deterministically correlated to *ε*_2_ by the well-established Kramer-Kronig equation^22^. Fundamentally, *ε*_2_ is related with interband transitions as^8^,^22^,^23^




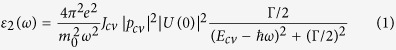




where *ω* is the frequency, *ħ* is the Planck’s constant, *e* and *m*_0_ are the charge and mass of free electrons, *J*_cv_ is the joint density of the initial (valence band) and final (conduction band) states involved in the transition. *p*_cv_ is an optical matrix element indicating the probability of the transition from the initial to final states. It consists of an integral over a unit cell that involves the momentum operator as well as the unit cell wavefunctions in the conduction and valence bands. 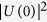 represents the effect of excitons on the oscillator strength of the interband transition, where *U* is the relative motion wavefunction of the eletrons and holes bound by Coulomb interactions and *0* indicates the physical overlap of the electron and hole wavefunctions. *E*_cv_ is the optical energy gap between the conduction and valence bands involved and *Γ* is a damping constant determining the bandwidth of the interband transition.

For simplicity, we only focus on the dielectric function at the peak position (on-resonance) as shown in Fig. 1c,d, where *E*_cv_ − *ħω* = 0 and eq.(1) can be simplified as




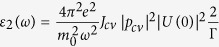




Physically, *Γ* represents the width of the peak. From Fig. 1b the peak can be found phenomenally remaining to be similar in the films with different layer numbers. Therefore, it is reasonable to consider that *Γ* is independent of the layer number. The optical matrix element *p*_cv_ is also independent of the layer number because it is only related with unit cells and unit cell wavefunctions, both of which are not dependent on the layer number. The independence of *p*_cv_ on geometrical features has previously been demonstrated at quantum wells^24^. Therefore, eq. (2) can be further simplified as




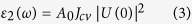




where *A*_0_ includes all the terms independent of the layer number. Eq. (3) indicates that the layer dependence of the dielectric function may result from only two parameters: the joint density of states *J*_cv_ and the excitonic effect 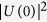.

We can get more insight by further examining the specific layer dependence of the two parameters *J*_cv_ and 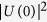. The joint density of states *J*_cv_ is determined by the band structure and expected to monotonically increase with the layer number. This is because the density of states in 2D materials is well known to be smaller than that in 3D materials^8^,^22^ and the increase of the layer number in effect enables a continuous evolution from two dimensions (monolayers) to three dimensions (bulk). To quantitatively elucidate the layer dependence of *J*_cv_, we calculate the *ε*_2_ of MoS_2_ films using density functional theory (DFT) techniques without considering excitonic effects^18^. The calculation result is given in Fig. 2a and essentially represents the density of states *J*_cv_. It reproduces the major spectral features of the experimental results and indeed shows a monotonic increase with the layer number. For the convenience of comparison, we plot the calculated and measured dielectric functions at the *C* peak as a function of the layer number in Fig. 2b. The calculated results are understandably smaller than the experimental results due to the exclusion of excitonic effects. Of our interest is to compare the layer dependence in the calculated and experimental results. The similarity of the two results for the films >5L suggests that the observed layer-dependent increase in the dielectric function in the relatively thicker films may be correlated to the effect of the density of states *J*_cv_. However, for the films less than 5 layers thick the calculated layer-dependence is opposite to the experimental observation (Fig. 2b). This can be correlated to the other parameter not considered in the calculation, the excitonic effect 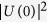. The excitonic effect is expected to be strong and to quickly decrease with the layer number due to the well known layer-dependent exponential decrease of exciton binding energy^15^.

The layer-dependent excitonic effect can be understood more quantitatively by examining the excitonic peak position with an intuitive model that involves quantum confinement and exciton binding energy. Of our interest is to quantitatively evaluate the layer dependence of the exciton binding energy from the observed evolution of the *C* exciton peak position with the layer number (Fig. 3a). The excitonic peak position (i.e. optical bandgap) is equal to the electronic bandgap minus the exciton binding energy, and its layer dependence originates from the layer dependence of both components. Should the layer dependence of the electronic bandgap be found out, we would be able to figure out the layer dependence of the exciton binding energy from the measured excitonic peak position. It is actually very difficult to experimentally or theoretically evaluate the electronic bandgap of MoS_2_ with different layer numbers. However, we find out that the layer dependence of the electronic bandgap may be reasonably correlated to quantum confinement effects. The peak position of the *C* exciton *E*_C_ in the films thicker than 7 layers shows a linear dependence on 1/*L*^2^ (Fig. 3b), and can be fitted by the conventional model of infinite quantum wells as *E*_C_ = *E*_g_ + *R*_y_ + *π*^2^*ħ*^2^/2*m*_eff_*L*^2^ – *R*_y_. where *E*_g_ is the position (optical bandgap) of the *C* peak in bulk MoS_2_ materials (*E*_g_ = 2.695 eV as measured in Fig. 1b), *R*_y_ is the exciton binding energy and assumed not changing with the thickness in the model of infinite quantum wells, and *m*_eff_ is the reduced electron-hole effective mass of the film. The introduction of *R*_y_ in the equation is to illustrate that the optical bandgap is equal to the electronic bandgap (the first three terms) minus the exciton binding energy (the last term). The fitting to the experimental results (the red lines in Fig. 3a,b) gives *E*_*C*_ = 2.695 eV + 150.9/*L*^2^, from which we can derive the reduced effective mass *m*_eff_ = 0.250 *m*_0_ for the *C* extions in bulk MoS_2_ and the films thicker than 7 layers. We can also derive the exciton binding energy *R*_*y*_ = 58.9 meV and Bohr radius *a*_b_ = 1.61 nm in bulk MoS_2_ and thick films, in which the static dielectric constant is set to be 7.6 as measured for bulk MoS_2_ previously^25^.

The peak position *E*_*C*_ in the films thinner than 5–7 layers shows apparent deviation from the model of infinite quantum wells (Fig. 3b). Instead, we can fit the experimental results with a model of quantum wells in fractional dimensional space as^26^,^27^




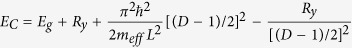




where *D* is the effective dimensionality that is defined by the ratio of the exciton binding energy *R*_y_^*^ in the films and that of bulk MoS_2_ *R*_*y*_ as [(*D* − 1)/2]^2^ = *R*_y_/ *R*_y_^*^. Again, the first three terms of eq. (4) represent the electronic bandgap and the last term indicates the exciton binding energy. The factor of [(*D* − 1)/2]^2^ in the third term originates from the change in the effective mass associated with the effective dimensionality. For the films thicker than 7 layers, *D* is 3 and eq. (4) is then reduced to the equation for infinite quantum wells. By fitting the experimental results with eq. (4), we can have the effective dimensionality *D* as 1.75, 2.07, 2.30, 2.51, 2.65, and 2.83 for the films in layer number of 1, 2, 3, 4, 5, and 6, respectively. We can then derive the corresponding exciton binding energies using *R*_y_^*^ = *R*_y_/[(*D* − 1)/2]^2^ as 0.421 eV, 0.206 eV, 0.139 eV, 0.103 eV, 0.0865 eV, and 0.0704 eV; we can also derive the corresponding Bohr radius of excitons from *a*_b_^*^ = *a*_b_(*D* − 1)/2^26^,^27^ as 0.602 nm, 0.861 nm, 1.04 nm,1.22 nm,1.33 nm, and 1.47 nm, respectively. These results are plotted in Fig. 3c.

The model we used to fit the experimental result is based on an assumption that the layer dependence of the electronic bandgap can be ascribed to the effect of quantum confinement. This is supported by our experimental results, in particular, the consistence between the observed peak position of the films thicker than 7 layers and what predicted from the model of infinite quantum wells. However, more theoretical and experimental studies would be necessary to provide more rigorous support, which is to our best knowledge expected to very difficult. Nevertheless, the result we obtained by fitting the experimental results using this model seems to be reasonable when compared to the limited number of studies on the exciton binding eerngy and Bohr radisu available in the literature. There is not study that would allow us to systematically crosscheck all of our results. For instance, the binding energy *R*_*y*_ = 58.9 meV and Bohr radius *a*_b_ = 1.61 we derived for the *C* exciton in bulk MoS_2_ and thick films is reasonably consistent with the binding energy and Bohr radius reported for the *A* exciton in bulk MoS_2_, which are 87.2 meV and 1.11 nm, respectively^28^. Additionally, the Bohr radius (0.602 nm) we derived for the *C* exciton in monolayer MoS_2_ nicely matches the theoretical prediction, ~0.5 nm^17^. The derived binding energy (0.421 eV) is reasonable compared with what reported for the *A* exciton, which is believed to be 0.4–0.6 eV in monolayer MoS_2_^18^,^29^.

With the information of the exciton binding energy and radius, the observed layer dependence of the dielectric function can be intuitively understood from a perspective of geometric confinement. Figure 4 shows the comparison between the size of excitons and the thickness of the film. While the film is highly anisotropic, the size of the exciton is schematically illustrated by the diameter of a sphere anyway. This is because eq. (4), which we used to derive the exciton radius, treats the excitons as spheres in an isotropic space by converting the geometrical anisotropy into fractional dimensionality^26^,^27^. The size of the exciton in bulk MoS_2_, 3.22 nm, is close to the thickness of the 5L film, 3.10 nm (Fig. 4a). Therefore, the exciton in MoS_2_ films is expected to start experiencing substantial geometrical confinement when the layer number of the film is decreased to 5, which may lead to decrease in the exciton size. Intuitively, a smaller exciton radius can better facilitate the spatial overlap of the electron and hole wavefunctions and subsequently cause larger amplitude in 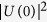. The layer-dependent decrease of the dielectric function is expected when the layer-dependent decrease of the excitonic effect offsets or even exceeds the layer-dependent increase of the join density of states.

The main conclusion we draw from the analysis of the *C* exciton, i.e. the dominance of excitonic effects in the dielectric function, can be applied to the *A* and *B* excitons as well due to the similar layer dependence in corresponding dielectric functions (Fig. 1c,d). However, it is difficult to quantitatively extract the binding energy and Bohr radius for the *A* and *B* excitons as what we did for the *C* exciton. This is because that the positions of the *A* and *B* excitons do not show substantial layer dependence as what observed as the *C* exciton, which makes the fitting by the intuitive model difficult. One reason for the less layer dependence observed at the *A* and *B* excitons could be related with the random variation of the position of the *A* and *B* excitons due to local doping effect from the substrate, which may be as large as ~10 meV^30^. Another reason could be related with the better localization of the A and B excitons in the plane of the film, which may lead to a less dependence on interlayer interactions^31^.

While our work mainly focuses on MoS_2_, we believe that similar dominance of excitonic effects in the dielectric function could generally exist in all the atomically thin semiconducting TMDC materials. Our result bears significant implications for the development of photonics devices with 2D TMDC materials. The obtained dielectric function and refractive index (Table S1) for the MoS_2_ with different layer numbers can be immediately useful for the rational design of photonic devices. In particular, as excitons are subject to influence of electric or magnetic fields, the dominance of excitonic effects in the dielectric function makes atomically MoS_2_ films an unprecedented platform that may enable the development of field-effect photonics, whose optical functionalities would be tuned by external electric or magnetic fields.

## Additional Information

**How to cite this article**: Yu, Y. *et al.* Exciton-dominated Dielectric Function of Atomically Thin MoS_2_ Films. *Sci. Rep.* **5**, 16996; doi: 10.1038/srep16996 (2015).

## Supplementary Material

Supplementary Information

## Figures and Tables

**Figure 1:**
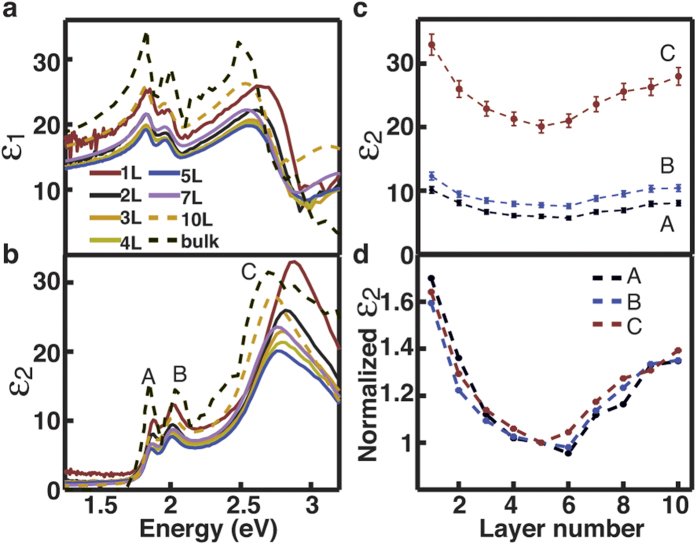
Anomalous layer-dependence of the dielectric function of 2D MoS_2_. (**a,b**) Real and imaginary parts of the dielectric function of 2D MoS_2_ vs. layer number. Also given is the dielectric function of bulk MoS_2_. The three peaks can be assigned to *A*, *B*, and *C* excitons as labeled. Corresponding refractive indexes of the films are given in Fig. S6 and Table S1 of the Supplementary Information. (**c**) The dependence of the imaginary part ε_2_ of the dielectric function at the *A*, *B*, and *C* peaks on layer number. The error bar is 5% and estimated from the measurement results of multiple samples. (**d**) Normalized *ε*_2_ at the *A*, *B*, and *C* peaks vs. layer number. The normalization is performed with respect to the corresponding value of each peak in the 5-layer MoS_2_. Error bar is ignored for visual convenience.

**Figure 2:**
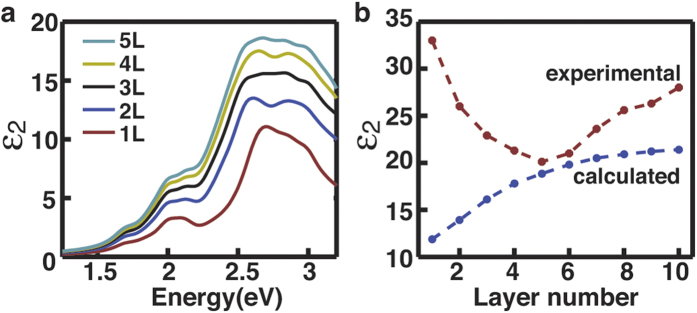
Comparison of the measured and calculated dielectric function of 2D MoS_2_. (**a**) Calculated imaginary part ε_2_ of the dielectric function of MoS_2_ with different thickness. (**b**) Comparison of experimental and calculated results for ε_2_ at the *C* peak as a function of the layer number. The error bar in the experimental result is ignored for visual convenience.

**Figure 3:**
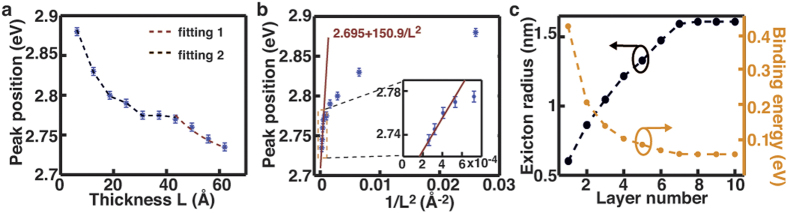
Strong, layer-dependent excitonic effects in atomically thin MoS_2_ films. (**a**) The position of the *C* peak in MoS_2_ films as a function of the thickness of the film *L*. The error bar ± 0.005 eV results from the possible errors in determining the peak position. The dashed lines are the fitting results using the model of infinite quantum wells (fitting 1) and the quantum well in fraction space (fitting 2) (**b**) The position of the *C* peak in MoS_2_ films as a function of 1/*L*^2^, where *L* is the thickness of the film. The red line is the fitting results using the model of infinite quantum well with the fitting equation given as shown. The inset is a magnified version of the area indicated by the dashed yellow rectangle. (**c**) The dependence of the binding energy and exciton radius in MoS_2_ films on the layer number.

**Figure 4:**
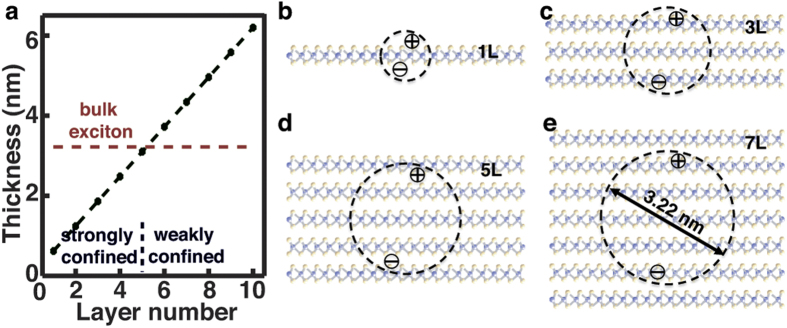
Geometric confinement of excitons in MoS_2_ films. (**a**) Comparison of the size of the exciton in bulk MoS_2_ with the thickness of MoS_2_ films. The regime where the film is thinner than the exciton size is categorized as strong confinement. (**b–e**) Schematic illustration of the size of excitons in the films with different layer numbers.
