# Supplementary material for: Exciton-dominated Dielectric Function of Atomically Thin MoS2 Films
Source: Sci Rep. 2015 Nov 24;5:16996. doi: 10.1038/srep16996 (PMC4657050; doi:10.1038/srep16996)
Supplement: Supplementary Information [file srep16996-s1.pdf]

Supplementary Information

Exciton-dominated Dielectric Function of Atomically Thin MoS_2 Films

Yiling Yu[2], Yifei Yu[1], Yongqing Cai[3], Wei Li[4], Alper Gurarslan[1,5], Hartwin Peelaers[6]
David E. Aspnes[2], Chris G. Van de Walle[6], Nhan V. Nguyen[4], Yong-Wei Zhang[3], Linyou Cao[1,2]*

[1]Department of Materials Science and Engineering, North Carolina State University, Raleigh, NC 27695, USA; [2]Department of Physics, North Carolina State University, Raleigh NC 27695, USA; [3] Institute of High Performance Computing, A*STAR, Singapore 138632; [4]Semiconductor and Dimensional Metrology Division, National Institute of Standards and Technology, Gaithersburg, Maryland 20899, USA; [5]Department of Fiber and Polymer Science, North Carolina State University, Raleigh, NC 27695, USA; [6]Materials Department, University of California, Santa Barbara, CA 93106, USA

* To whom correspondence should be addressed.
Email: lcao2@ncsu.edu

This PDF file includes
Methods
Fig. S1-S12
Table. S1 Tabulated refractive index of MoS_2 films
References S1-S4

Methods

Synthesis of centimeter-scale MoS_2 films. The centimeter monolayer and fewlayer MoS_2 films were grown using a chemical vapor deposition (CVD) process that we have previously developed[1]. In a typical growth, 4-20 mg molybdenum chloride ($MoCl_5$) powder (99.99%, Sigma-Aldrich) and 1g sulfur powder (Sigma-Aldrich) was placed at the upstream of a tube furnace. Receiving substrates (typically sapphire) were placed in the downstream of the tube. Other typical experimental conditions for the growth include a temperature of 850 °C, a flow rate of 50 sccm, and a pressure around 2 Torr. The layer number was controlled by controlling the amount of precursors ($MoCl_5$) used in the synthesis. We also grow centimeter-scale monolayer MoS_2 films using a similar chemical vapor deposition process but with MoO_3 and sulfur being used as precursors

The composition and structure of the synthesized MoS_2 films were extensively characterized by a variety of tools previously. In this work we mainly focused on characterizing the thickness and surface morphology of the synthesized films with optical microscopes and atomic force microscope (AFM, Veeco Dimension-3000).

Ellipsometry measurements of MoS_2 films. The ellipsometry measurement was performed with Woollam VASE (Variable Angle Spectroscopic Ellipsometer, J.A. Woollam Co.) with a Xenon light source in range of 200-1100nm. Typical incident angle was set at 65^0, but we confirmed that the incident angle does not affect the resulting dielectric constants. The ellipsometry measurement essentially monitors changes in the polarization state of incident light and the light reflected from the films. It yields two spectral parameters (ψ and Δ) related with the amplitude (tan ψ) and phase Δ of a reflectance ratio ρ, which indicates the ratio of the reflection coefficients for *p*-polarized (parallel to the plane of incidence) and *s*-polarized (perpendicular to the plane of incidence) light, $\rho = r_p/r_s = (\tan\psi)e^{i\Delta}$. To retrieve the optical constant from the measured results, we perform regression fitting using the Fresnel's equations of a simple two-layer model that consists of a MoS_2 film on top of a semi-infinite substrate. Precise information of the thickness of the film is required for the fitting, which we have obtained by AFM measurements in experiments. We have also confirmed that the surfaces of all the films studied are atomically smooth (roughness < 1 nm) by the AFM measurements (Fig. S1-S3). The typical fitting for experimental results is given in Fig. S4.

First-principles calculations. Our DFT calculations are carried out using the plane wave code Vienna ab initio simulation package (VASP)[2] with the generalized gradient approximation (GGA). Spin-orbital coupling calculations using the projector augmented wave method with the Perdew–Burke–Ernzerhof functional (PAW-PBE) and a cutoff energy of 400 eV are performed. The multilayer MoS_2 structures are adopted by the experimental lattice of bulk 2H-MoS_2, and the structures are fully relaxed until the Hellmann-Feynman forces become less than 0.01 eV/Å, by using the van der Waals optB88 functional[3] to obtain an accurate description of the dispersion force and the interlayer distance. The first Brillouin zone is sampled with a $15\times15\times1$ Monkhorst-Pack grid and a vacuum region with thickness greater than 15 Å is adopted.

The optical properties are calculated based on independent-particle approximation. The involved unoccupied band number is about 10 times of that of valence bands to achieve the converged dielectric function. The accuracy of the k-point sampling is tested for using 15×15 and 18×18 grid for 1L-3L MoS_2, and the calculated dielectric spectra is shown in Fig. S7. While there are

some discrepancies in the low-energy part between these two k-points sets, good convergence is observed for the "C" peak at around 2.75 eV, consistent with previous work[4]. Similarly, for the SOC effect (Fig. S8), the imaginary part of the dielectric constant for MoS_2 layers from 1L to 5L shows little effect from the SOC.

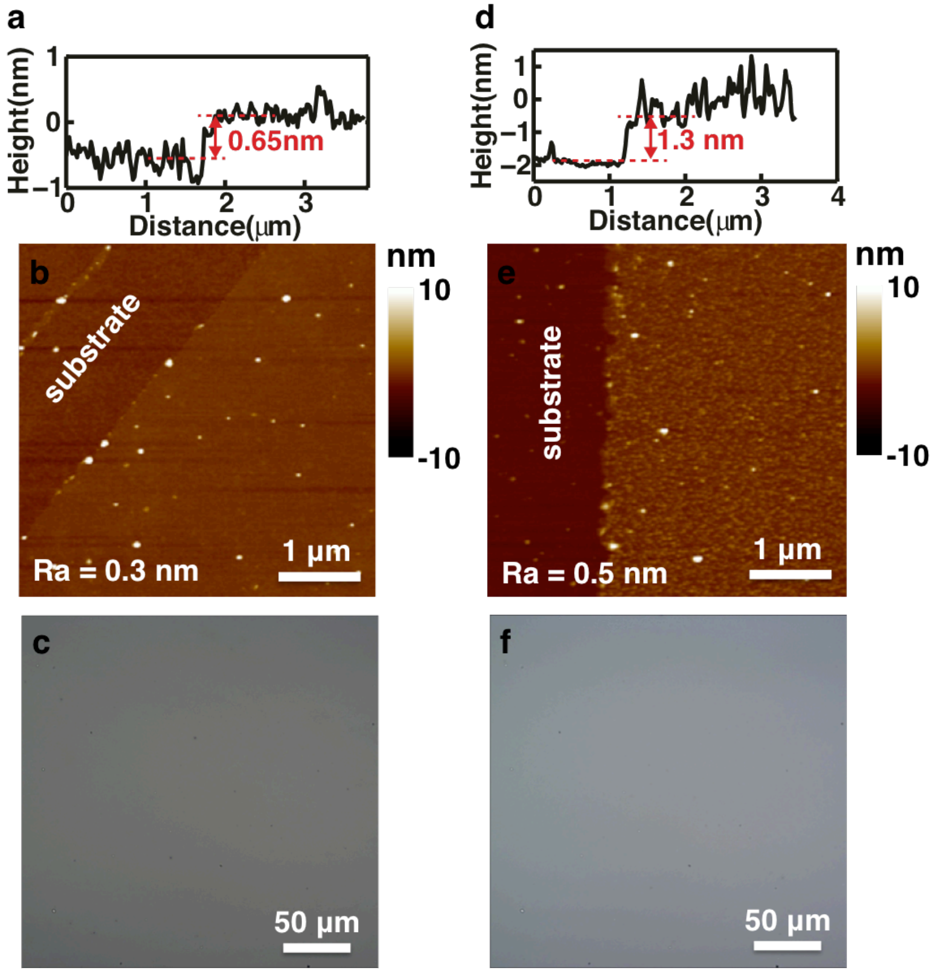

Figure S1. Characterization of monolayer and bilayer MoS2 films. (a-c) Typical height profile extracted from AFM measurements, AFM image, and optical image of monolayer MoS_2 films. The surface roughness is measured as 0.3 nm as shown in the AFM image. **(d-f)** Typical height profile extracted from AFM measurements, AFM image, and optical image of bilayer MoS_2 films. The surface roughness is measured as 0.5 nm as shown in the AFM image.

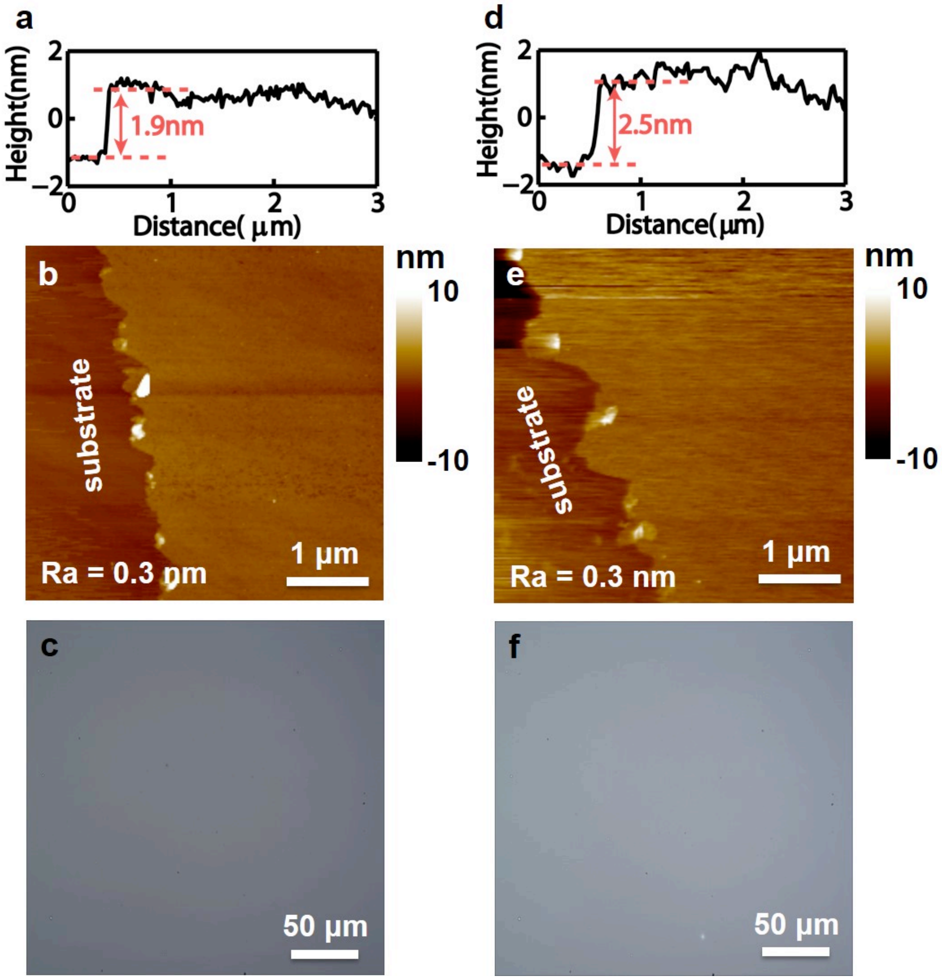

Figure S2. Characterization of three-layer (3L) and four-layer (4L) MoS_2 films. (a-c) Typical height profile extracted from AFM measurements, AFM image, and optical image of 3L MoS_2 films. The surface roughness is measured as 0.3 nm as shown in the AFM image. **(d-f)** Typical height profile extracted from AFM measurements, AFM image, and optical image of 4L MoS_2 films. The surface roughness is measured as 0.3 nm as shown in the AFM image.

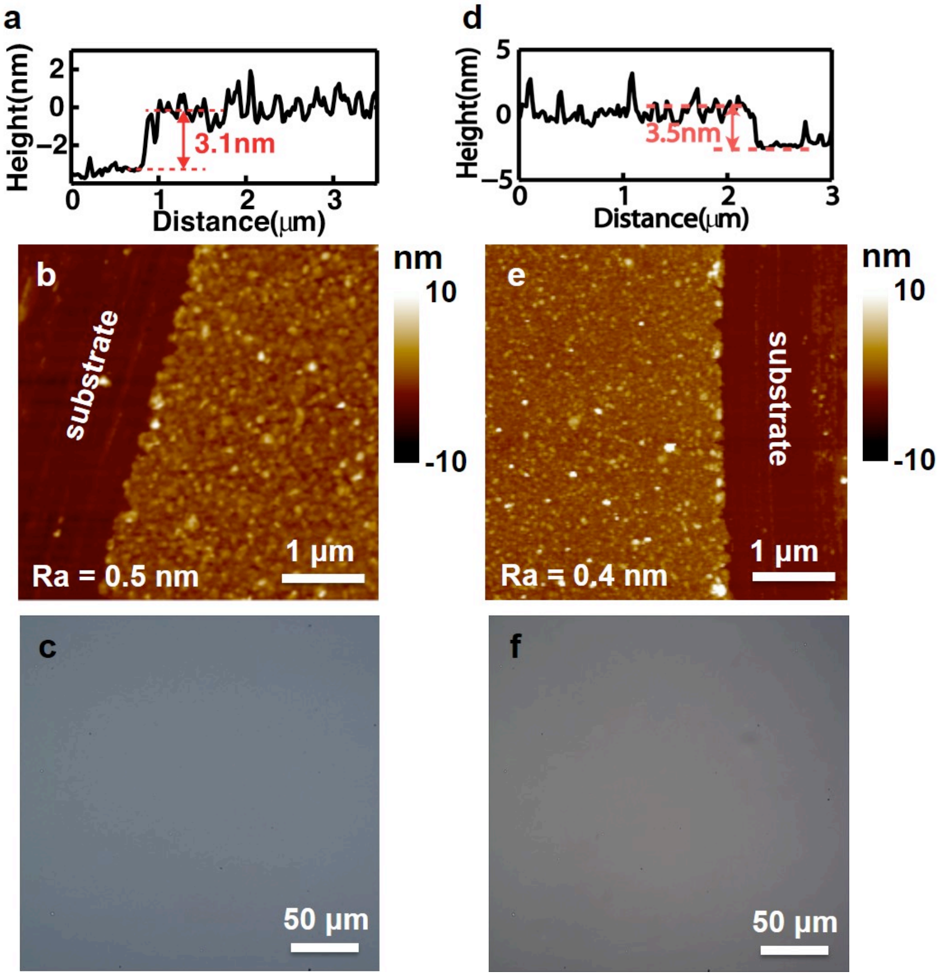

Figure S3. Characterization of five-layer (5L) and six-layer (6L) MoS_2 films. (a-c) Typical height profile extracted from AFM measurements, AFM image, and optical image of 5L MoS_2 films. The surface roughness is measured as 0.5 nm as shown in the AFM image. **(d-f)** Typical height profile extracted from AFM measurements, AFM image, and optical image of 6L MoS_2 films. The surface roughness is measured as 0.4 nm as shown in the AFM image.

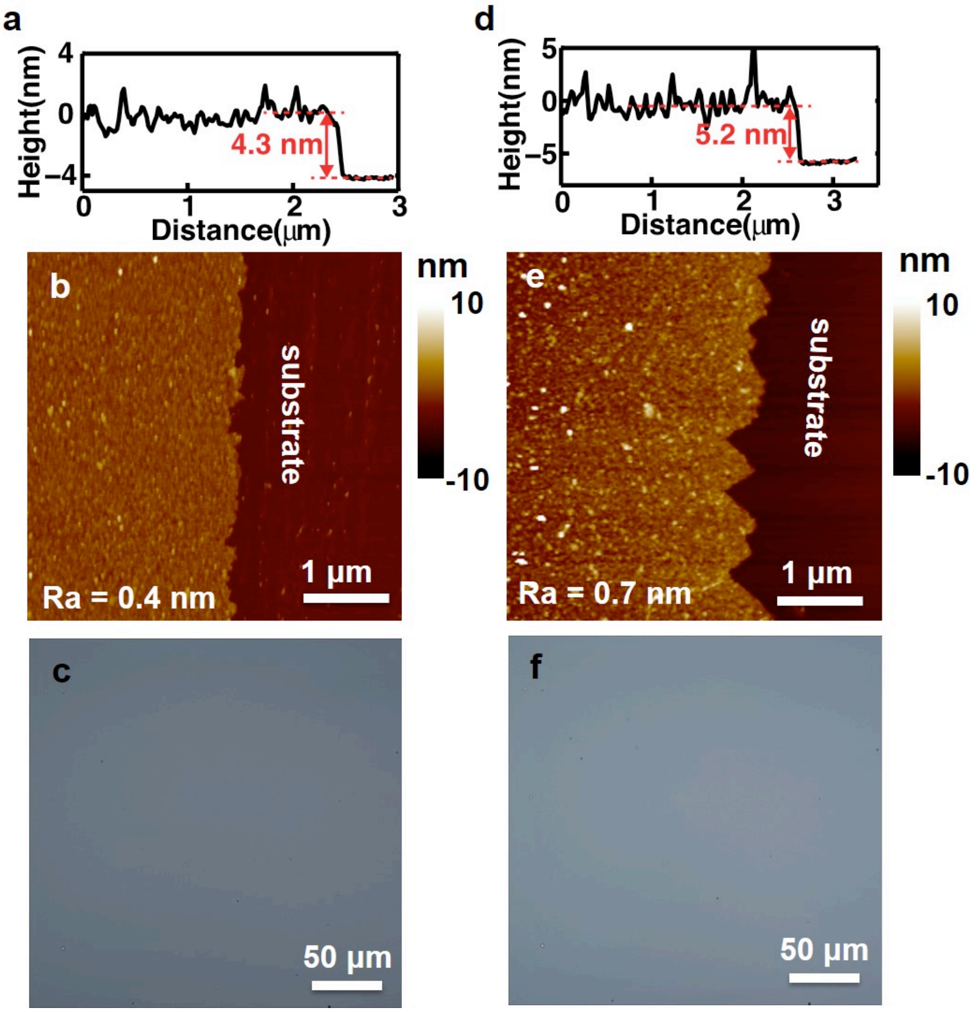

Figure S4. Characterization of seven-layer (7L) and eight-layer (8L) MoS_2 films. (a-c) Typical height profile extracted from AFM measurements, AFM image, and optical image of 7L MoS_2 films. The surface roughness is measured as 0.4 nm as shown in the AFM image. **(d-f)** Typical height profile extracted from AFM measurements, AFM image, and optical image of 8L MoS_2 films. The surface roughness is measured as 0.9 nm as shown in the AFM image

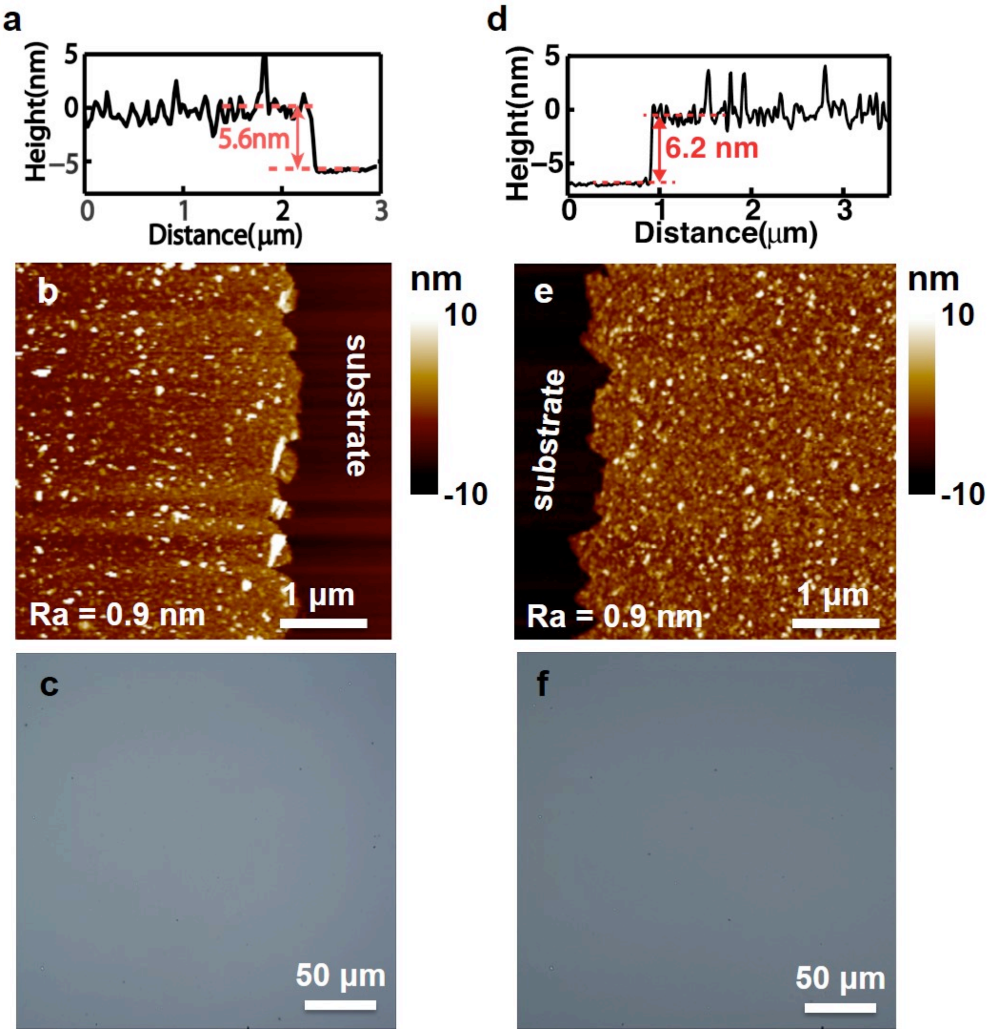

Figure S5. Characterization of nine-layer (9L) and ten-layer (10L) MoS_2 films. (a-c) Typical height profile extracted from AFM measurements, AFM image, and optical image of 9L MoS_2 films. The surface roughness is measured as 0.9 nm as shown in the AFM image. **(d-f)** Typical height profile extracted from AFM measurements, AFM image, and optical image of 10L MoS_2 films. The surface roughness is measured as 0.9 nm as shown in the AFM image

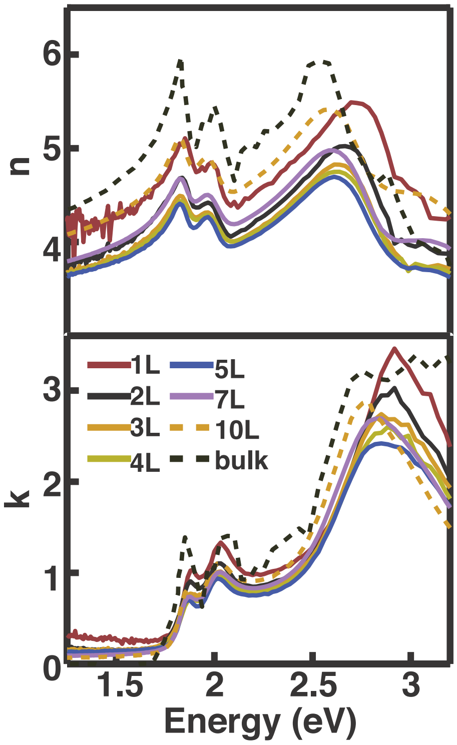

Figure S6. Refractive index of 2D MoS_2. Real (upper panel) and imaginary (lower panel) parts of the dielectric function of 2D MoS_2 vs. layer number. Also given is the refractive index of bulk MoS_2.

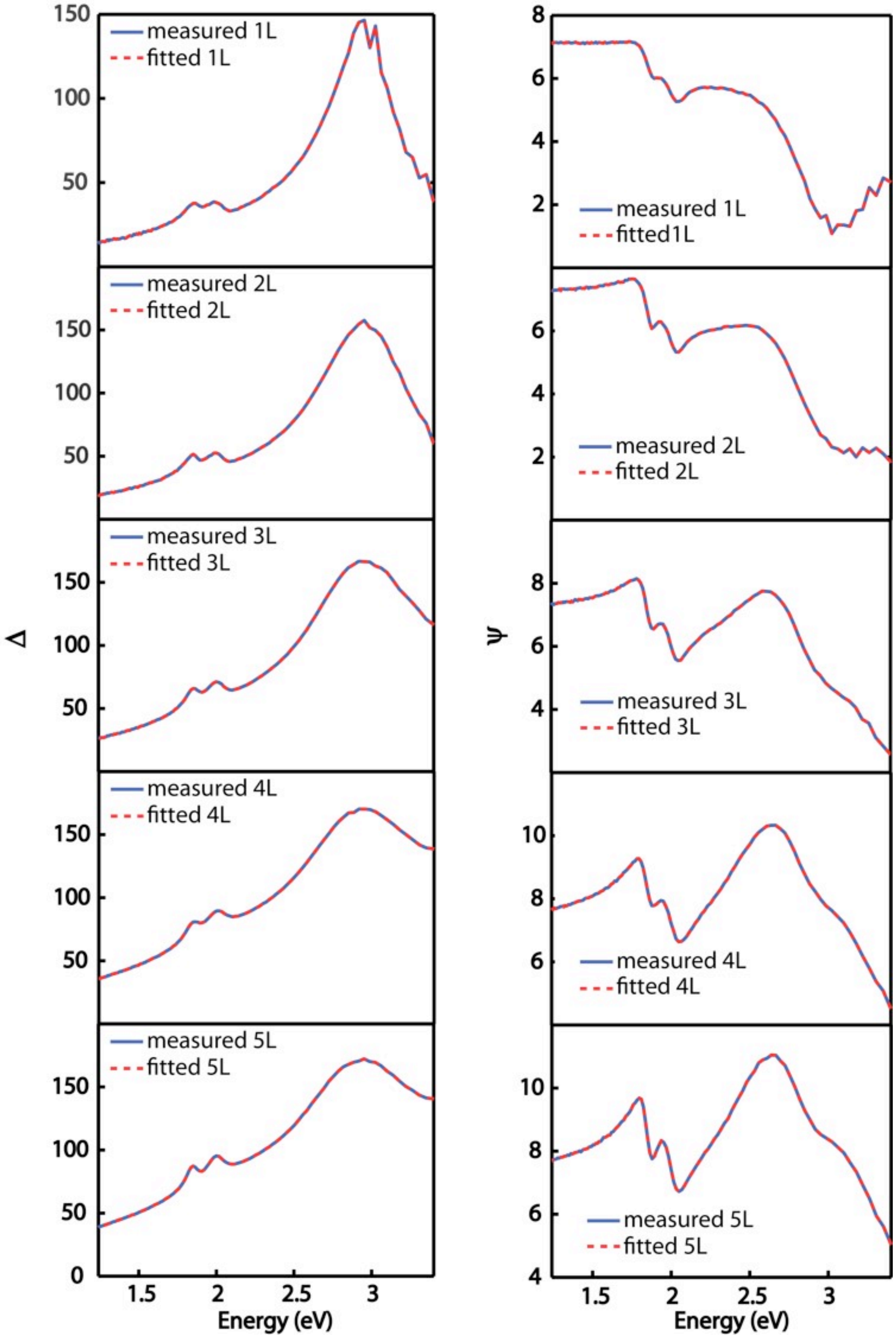

Figure S7. Fitting for the results obtained from ellipsometry measurements. (a) Fitted and measured phase Δ from monolayer to 5-layer MoS2 films. (b) Fitted and measured amplitude ψ for monolayer to 5-layer MoS2 films.

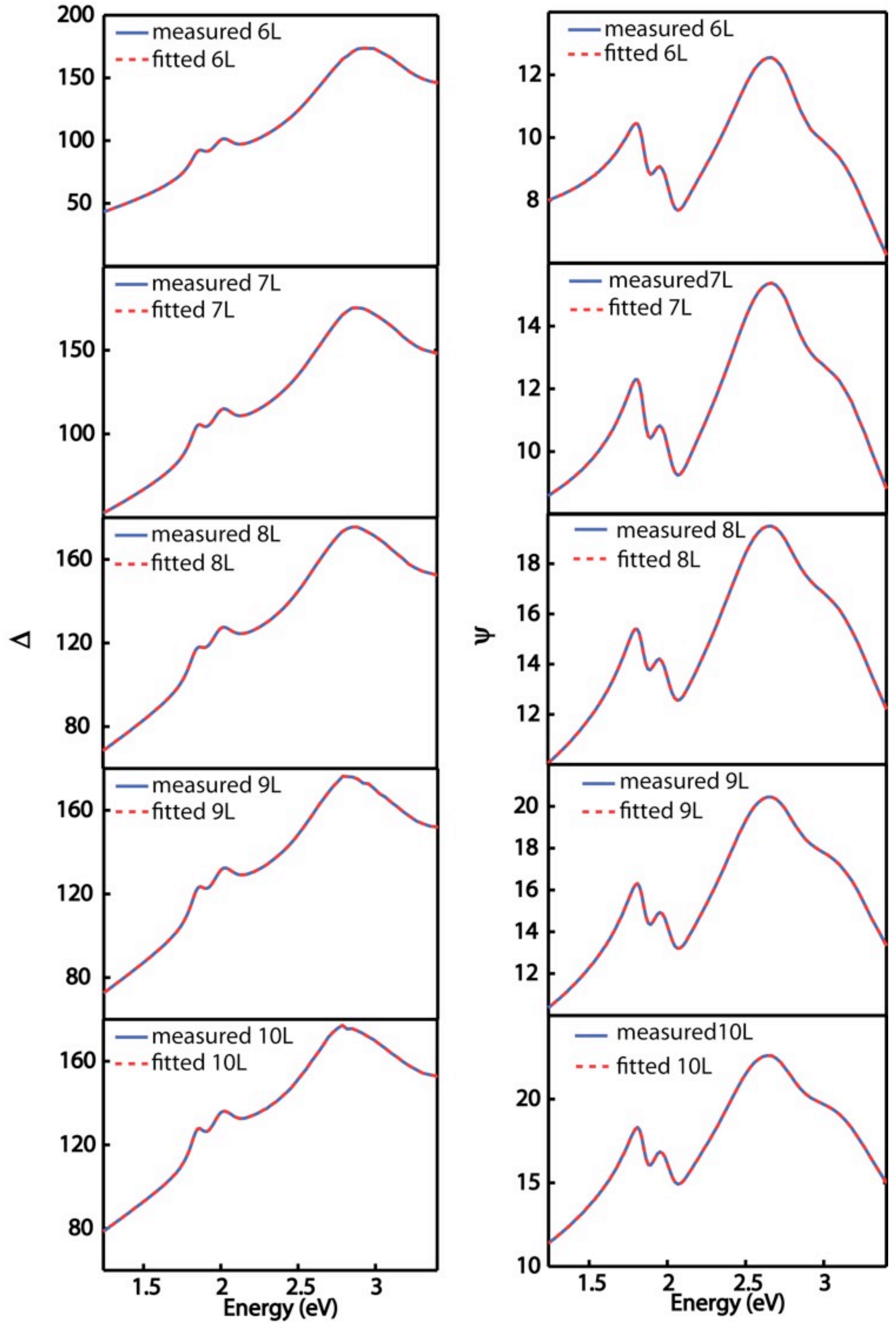

Figure S8. Fitting for the results obtained from ellipsometry measurements. (a) Fitted and measured phase *Δ* from 6-layer to 10-layer MoS2 films. (b) Fitted and measured amplitude *ψ* for 6-layer to 10-layer MoS2 films.

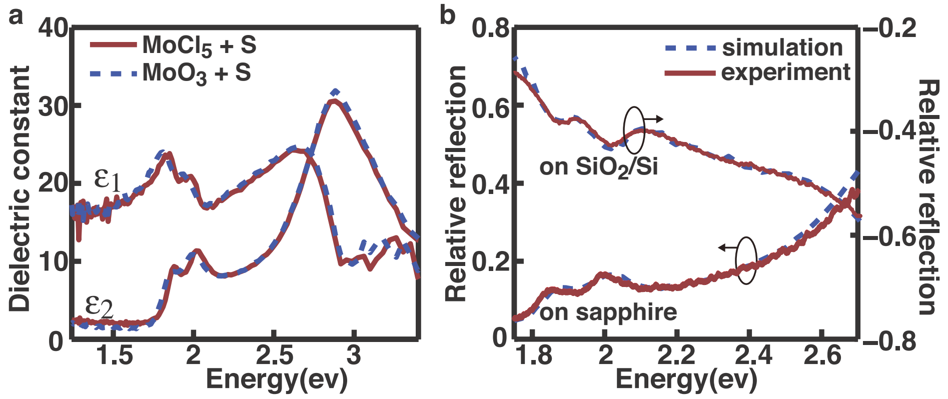

Figure S9. Independence of the measured dielectric function from synthetic processes and substrates. (a) Measured dielectric constants of as-grown monolayer MoS_2 made by two different CVD processes, one using $MoCl_5$ and S as the precursors and the other using MoO_3 and S as the precursors. The growth substrate in both growths is sapphire. (b) Measured and simulated reflection spectra of monolayer MoS_2 on different substrates, sapphire and silicon with 80 nm thick thermal oxide. The monolayer MoS_2 involved is grown on sapphire substrates and then transferred to SiO_2/Si substrates. The simulation uses the optical constant measured with the as-grown MoS_2 on sapphire substrates.

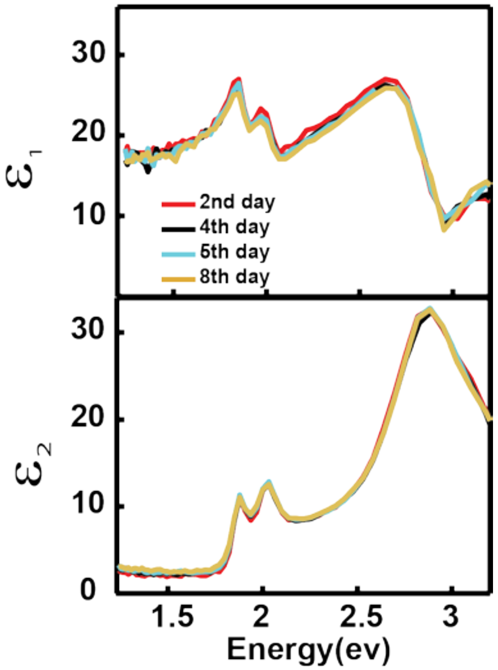

Figure S10. Stability of the dielectric function. Real (upper panel) and imaginary (lower panel) parts of the dielectric function of monolayer MoS_2 measured at different times after synthesis.

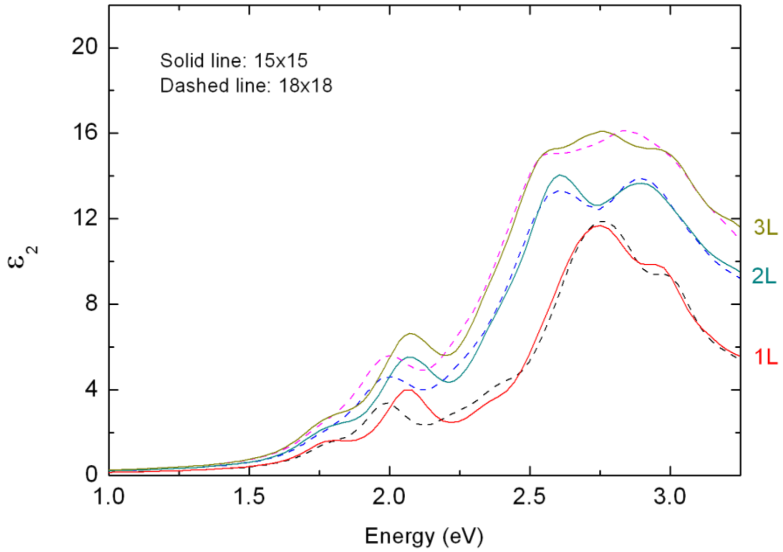

Figure S11. Imaginary part of the dielectric constant for MoS_2 layers from 1L to 3L calculated different k-point sampling.

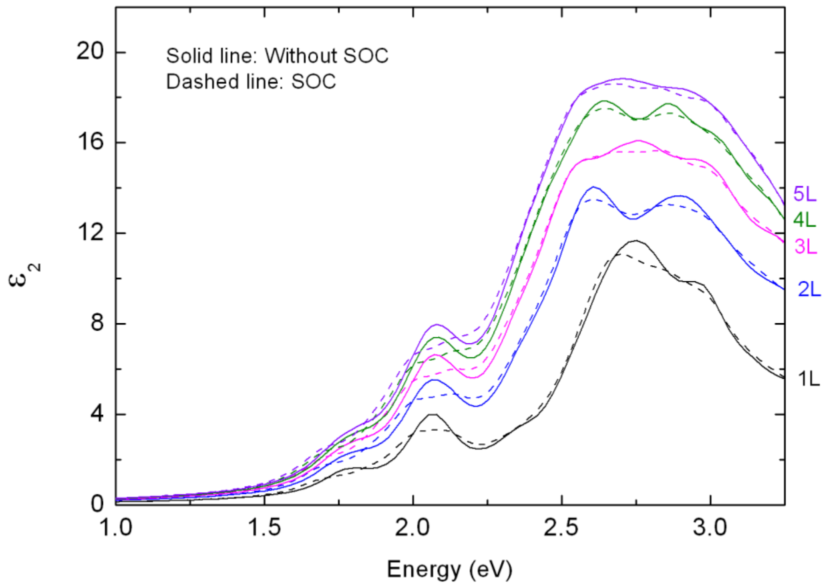

Figure S12. Imaginary part of the dielectric constant for MoS_2 layers from 1L to 5L calculated with spin-orbital coupling (dashed lines) and without spin-orbital coupling (solid lines).

Table S1. Tabulated refractive index of MoS_2 films

	1L		2L		3L	
wavelength(nm)	n	k	n	k	n	k
345	3.035	2.1095	3.1215	1.48	3.1584	1.4242
350	3.3642	1.711	3.1431	1.6304	3.1799	1.3707
355	3.2948	1.7682	3.3188	1.5147	3.1516	1.4211
360	3.0747	2.2111	3.4153	1.4939	3.3381	1.3626
365	3.4136	1.9955	3.482	1.4957	3.3745	1.4535
370	4.0257	1.8186	3.5141	1.636	3.4953	1.5307
375	3.9504	1.964	3.6118	1.6953	3.5514	1.6638
380	4.2656	2.1141	3.7339	1.8568	3.6016	1.8068
385	4.2966	2.2502	3.8891	1.9414	3.6983	1.8775
390	4.241	2.5232	3.8747	2.128	3.7274	1.9909
395	4.2479	2.7103	3.8809	2.3194	3.7528	2.1365
400	4.2558	2.9601	3.9263	2.4171	3.7368	2.2653
405	4.4645	2.9673	3.987	2.4962	3.721	2.4118
410	4.538	3.1094	3.9502	2.6409	3.7648	2.4578
415	4.5785	3.2506	3.9717	2.7826	3.7127	2.6254
420	4.6688	3.3453	4.0257	2.8704	3.816	2.6231
425	4.6942	3.461	3.9825	3.0278	3.889	2.6862
430	4.9523	3.334	4.2523	2.9482	4.0043	2.6842
435	5.1659	3.1845	4.3822	2.9453	4.0705	2.7412
440	5.3321	2.9496	4.5641	2.8467	4.2579	2.6839
445	5.4295	2.7198	4.724	2.6996	4.4327	2.5853
450	5.471	2.5039	4.8808	2.5191	4.5802	2.4602
455	5.4828	2.3085	4.9702	2.3155	4.6858	2.2951
460	5.4897	2.1087	5.0077	2.128	4.7639	2.1338
465	5.4255	1.9334	5.0223	1.9605	4.8122	1.9741
470	5.3851	1.7774	5.0186	1.7949	4.8247	1.802
475	5.3511	1.6549	4.9916	1.648	4.8261	1.6554
480	5.2685	1.5181	4.9396	1.509	4.7934	1.5116
485	5.1999	1.4334	4.9015	1.3921	4.7569	1.3899
490	5.1296	1.3522	4.8236	1.2948	4.7065	1.2803
495	5.0832	1.2878	4.7654	1.2128	4.6505	1.1969
500	5.0093	1.2361	4.7054	1.1513	4.5976	1.1257
505	4.9526	1.1885	4.6687	1.0974	4.5475	1.0737
510	4.918	1.1623	4.6115	1.0532	4.4963	1.0202
515	4.8614	1.1215	4.5572	1.0157	4.4548	0.97899
520	4.8404	1.0768	4.511	0.98054	4.4037	0.93817
525	4.7757	1.0698	4.4642	0.94246	4.354	0.91249
530	4.7458	1.0402	4.4245	0.92324	4.3213	0.89069
535	4.6875	1.0243	4.3847	0.91436	4.2826	0.87048

540	4.6276	1.0169	4.3481	0.8949	4.2396	0.85457
545	4.6184	1.0045	4.3139	0.87514	4.2103	0.84473
550	4.5974	0.98786	4.2807	0.86571	4.1887	0.83567
555	4.5692	0.97655	4.2529	0.8587	4.151	0.82515
560	4.5375	0.98979	4.2331	0.84906	4.1273	0.81697
565	4.4953	0.97569	4.1915	0.84723	4.1036	0.81305
570	4.4593	0.98802	4.1599	0.85819	4.0785	0.8257
575	4.4652	0.99232	4.1311	0.86358	4.0557	0.83162
580	4.4046	1.0283	4.1233	0.86556	4.0351	0.84601
585	4.3665	1.0652	4.0993	0.90042	4.0186	0.85957
590	4.4281	1.1133	4.0773	0.92505	4.0076	0.88298
595	4.3854	1.1736	4.0568	0.98182	4.007	0.91261
600	4.4185	1.2453	4.0802	1.0272	4.0194	0.95695
605	4.4832	1.293	4.1333	1.0777	4.0487	0.98958
610	4.5949	1.3319	4.1995	1.1032	4.1102	1.0138
615	4.7645	1.2887	4.2755	1.1024	4.1728	1.0085
620	4.823	1.2336	4.3632	1.0654	4.238	0.97982
625	4.8473	1.1104	4.405	0.99226	4.2943	0.9388
630	4.8233	1.0397	4.4202	0.93166	4.3156	0.8626
635	4.7608	0.9785	4.4086	0.87284	4.3128	0.80623
640	4.7993	0.9588	4.3911	0.84114	4.295	0.76433
645	4.757	0.94393	4.3689	0.82963	4.2724	0.7384
650	4.7445	0.97564	4.3675	0.84836	4.2619	0.74509
655	4.8177	1.033	4.3777	0.87778	4.2712	0.76408
660	4.9334	1.0246	4.448	0.90742	4.3095	0.77144
665	5.0486	0.94987	4.5632	0.87405	4.373	0.75421
670	5.1062	0.80879	4.6477	0.78976	4.4384	0.70404
675	5.0349	0.67168	4.6851	0.66598	4.4861	0.62352
680	5.0504	0.53835	4.6786	0.54976	4.4922	0.52238
685	4.9719	0.45368	4.6382	0.47019	4.4656	0.42973
690	4.9161	0.39993	4.5794	0.39449	4.4248	0.365
695	4.8819	0.34722	4.5294	0.34297	4.3719	0.31735
700	4.7792	0.31574	4.4932	0.30126	4.3385	0.28054
705	4.7406	0.3121	4.4461	0.27104	4.2891	0.2527
710	4.7011	0.3007	4.4192	0.24924	4.2509	0.23223
715	4.6449	0.28574	4.3796	0.21906	4.2112	0.21784
720	4.6281	0.2684	4.3454	0.20235	4.1777	0.20581
725	4.6008	0.25389	4.3018	0.18963	4.149	0.19284
730	4.5917	0.26534	4.2665	0.18329	4.1269	0.18748
735	4.564	0.25114	4.2493	0.17875	4.1062	0.18071
740	4.4731	0.25553	4.2084	0.17008	4.0695	0.1786
745	4.4781	0.23703	4.1905	0.16271	4.0515	0.17313
750	4.4968	0.26643	4.1663	0.1677	4.0331	0.16169
755	4.4555	0.24217	4.1474	0.16509	4.0284	0.16304

760	4.4546	0.25444	4.1122	0.15967	4.0049	0.1699
765	4.4104	0.25882	4.108	0.15712	3.9894	0.1553
770	4.3872	0.25604	4.0846	0.15634	3.9607	0.1585
775	4.3246	0.2661	4.0686	0.16126	3.9767	0.16305
780	4.3012	0.22656	4.0303	0.15816	3.9409	0.1541
785	4.3975	0.27913	4.0053	0.16128	3.9261	0.15155
790	4.3033	0.23178	4.0308	0.15668	3.8957	0.1591
795	4.3539	0.25401	3.9956	0.15633	3.882	0.16254
800	4.3375	0.2661	3.9946	0.1528	3.8753	0.14879
805	4.2829	0.27118	3.9805	0.15312	3.8912	0.15733
810	4.2618	0.21999	3.963	0.14757	3.871	0.14949
815	4.4143	0.24665	3.9551	0.15666	3.8526	0.15579
820	4.262	0.25912	3.9322	0.16006	3.8459	0.16055
825	4.2599	0.25859	3.9248	0.15353	3.837	0.15222
830	4.2536	0.28422	3.9241	0.14709	3.843	0.15066
835	4.3063	0.27486	3.9432	0.1558	3.8332	0.14969
840	4.2071	0.26591	3.9436	0.15872	3.8175	0.15397
845	4.0152	0.30428	3.9045	0.15062	3.8431	0.14614
850	4.384	0.28634	3.9846	0.16584	3.8452	0.17482
855	4.2731	0.27395	3.9277	0.16079	3.7805	0.1484
860	4.2313	0.29451	3.9019	0.16303	3.8043	0.16181
865	4.0518	0.25802	3.8836	0.16324	3.8042	0.16507
870	4.2107	0.29103	3.8418	0.159	3.7678	0.15209
875	4.1901	0.28231	3.8546	0.15625	3.7828	0.14107
880	4.2146	0.2688	3.8225	0.15519	3.7643	0.16109
885	4.1368	0.26439	3.8191	0.15187	3.7538	0.14589
890	4.2505	0.27241	3.8828	0.15811	3.7618	0.14685
895	4.1246	0.275	3.823	0.15444	3.7427	0.15058
900	4.2324	0.27653	3.8394	0.16086	3.7517	0.15551
905	4.1399	0.29945	3.8437	0.15368	3.7459	0.1452
910	4.0882	0.27867	3.8041	0.14879	3.7418	0.16137
915	4.204	0.27915	3.8372	0.16322	3.7354	0.15593
920	4.2151	0.29217	3.7608	0.1558	3.7179	0.14731
925	4.1818	0.31775	3.8349	0.16625	3.7208	0.15206
930	3.9989	0.27231	3.8169	0.17591	3.727	0.14932
935	4.2799	0.28077	3.8124	0.16884	3.6963	0.15023
940	4.2274	0.30284	3.7828	0.16941	3.6996	0.16622
945	4.3511	0.29421	3.7913	0.16523	3.7445	0.15839
950	4.2508	0.29206	3.7844	0.17303	3.6923	0.15044
955	4.3307	0.29048	3.8152	0.16167	3.7089	0.15293
960	4.1119	0.36195	3.7906	0.17346	3.6594	0.15831
965	3.6847	0.30302	3.7358	0.16777	3.6834	0.16439
970	4.2891	0.30635	3.7615	0.17787	3.6761	0.15848
975	4.2642	0.32016	3.7736	0.18801	3.7132	0.16278

980	4.0643	0.30154	3.7598	0.16946	3.6674	0.15559
985	4.0738	0.30023	3.7561	0.18424	3.6723	0.15389
990	4.26	0.31443	3.8044	0.17347	3.68	0.15773
995	4.1482	0.32433	3.7509	0.18282	3.6638	0.15621
1000	4.1655	0.32855	3.7309	0.1854	3.6428	0.15677

	**4L**		**5L**		**6L**	
wavelength(nm)	n	k	n	k	n	k
345	2.5614	1.8298	3.0358	1.3321	3.0844	1.3501
350	3.194	1.3347	3.0551	1.3281	3.1263	1.3278
355	3.125	1.4035	3.1296	1.3226	3.1888	1.3263
360	3.2429	1.4139	3.2271	1.321	3.2635	1.3337
365	3.2509	1.5094	3.2787	1.3537	3.3504	1.3692
370	3.4527	1.4604	3.3782	1.4042	3.4356	1.4218
375	3.5296	1.5183	3.4467	1.4665	3.5215	1.4918
380	3.5911	1.6489	3.5307	1.572	3.598	1.5798
385	3.6367	1.7441	3.5988	1.6661	3.6692	1.6879
390	3.694	1.8858	3.6456	1.7875	3.7171	1.8181
395	3.7279	2.0512	3.6812	1.9205	3.7502	1.9475
400	3.7228	2.1625	3.7	2.0316	3.7609	2.0817
405	3.7459	2.205	3.7055	2.1543	3.7696	2.1851
410	3.7663	2.3166	3.7282	2.2037	3.7707	2.2849
415	3.689	2.5041	3.716	2.311	3.7685	2.3836
420	3.8204	2.4765	3.7435	2.373	3.8037	2.4242
425	3.825	2.5621	3.8057	2.3871	3.8434	2.4684
430	3.9221	2.6016	3.8774	2.4095	3.9145	2.5102
435	4.0918	2.5361	3.9761	2.4199	4.025	2.5045
440	4.2156	2.5281	4.0915	2.4114	4.1697	2.4642
445	4.3628	2.4465	4.2231	2.3754	4.3019	2.4399
450	4.4941	2.3408	4.363	2.3038	4.4553	2.3357
455	4.6071	2.1983	4.4893	2.1779	4.5806	2.2191
460	4.6802	2.0611	4.584	2.0393	4.678	2.0717
465	4.7282	1.8988	4.6426	1.8974	4.7455	1.9145
470	4.749	1.7469	4.6799	1.7471	4.7787	1.762
475	4.7481	1.6091	4.696	1.5934	4.789	1.619
480	4.7362	1.4717	4.6796	1.4624	4.7778	1.4747
485	4.7031	1.3579	4.6507	1.3532	4.7532	1.3492
490	4.6591	1.2501	4.6138	1.2294	4.7125	1.2375
495	4.6139	1.1679	4.57	1.156	4.6664	1.145
500	4.5608	1.0933	4.5203	1.0741	4.614	1.068
505	4.5105	1.0365	4.4705	1.0094	4.562	1.0016
510	4.4681	0.98793	4.4263	0.95779	4.5105	0.94978
515	4.4167	0.94254	4.3782	0.91445	4.4636	0.90615

520	4.3717	0.91075	4.337	0.87585	4.416	0.86924
525	4.3385	0.87681	4.2945	0.85184	4.3725	0.83965
530	4.2953	0.85398	4.2561	0.82294	4.3307	0.81397
535	4.2566	0.83392	4.2181	0.80078	4.2934	0.79583
540	4.2195	0.82141	4.1836	0.78992	4.2575	0.77728
545	4.196	0.803	4.1526	0.7659	4.2234	0.76694
550	4.1629	0.79384	4.1246	0.76979	4.1946	0.75332
555	4.1337	0.79206	4.0911	0.75762	4.165	0.74949
560	4.1085	0.78915	4.0697	0.75156	4.1388	0.74522
565	4.0855	0.78346	4.0452	0.75376	4.1148	0.74631
570	4.0621	0.78814	4.0281	0.75084	4.0906	0.74932
575	4.0469	0.79079	4.0067	0.76026	4.0728	0.75755
580	4.0238	0.80428	3.9861	0.76684	4.0539	0.7701
585	4.0138	0.81894	3.9703	0.77828	4.0419	0.78739
590	4.0034	0.84196	3.9569	0.79617	4.0334	0.81297
595	4.0026	0.87146	3.9538	0.8258	4.0393	0.8412
600	4.0203	0.91388	3.9554	0.8607	4.0595	0.87293
605	4.0568	0.94228	3.9835	0.90045	4.0972	0.89817
610	4.1131	0.95913	4.0276	0.92813	4.1509	0.9087
615	4.1722	0.94849	4.1008	0.93788	4.2138	0.89697
620	4.2366	0.91014	4.1759	0.91607	4.2678	0.85902
625	4.272	0.85979	4.2334	0.85543	4.3055	0.80191
630	4.287	0.79769	4.2669	0.78154	4.3149	0.74104
635	4.2841	0.74063	4.2544	0.70846	4.3088	0.68818
640	4.2645	0.70184	4.2269	0.65469	4.2849	0.65194
645	4.2443	0.69144	4.1877	0.63311	4.2652	0.638
650	4.2341	0.69161	4.1576	0.6314	4.2554	0.64115
655	4.2472	0.69864	4.1489	0.65775	4.2645	0.65279
660	4.2836	0.70641	4.1709	0.69372	4.3021	0.65914
665	4.3376	0.68971	4.2397	0.70046	4.3599	0.64246
670	4.4007	0.63422	4.3282	0.67378	4.4217	0.58933
675	4.431	0.55125	4.3912	0.58729	4.4573	0.50454
680	4.4362	0.46543	4.4087	0.47751	4.4554	0.41062
685	4.4055	0.37922	4.3839	0.37166	4.4243	0.32837
690	4.3647	0.32056	4.3318	0.30779	4.378	0.26886
695	4.3169	0.27772	4.2695	0.25896	4.3291	0.22579
700	4.2732	0.24239	4.2228	0.22526	4.2818	0.19555
705	4.2329	0.21952	4.1818	0.20342	4.2399	0.17523
710	4.197	0.20274	4.1423	0.19703	4.2011	0.16097
715	4.1634	0.19636	4.1053	0.18286	4.1676	0.15099
720	4.1348	0.18719	4.0859	0.17904	4.1392	0.14232
725	4.1079	0.17553	4.0524	0.17421	4.1128	0.13598
730	4.0839	0.17365	4.0299	0.16416	4.0894	0.13149
735	4.0672	0.16776	4.0156	0.16941	4.067	0.12855

740	4.0394	0.15977	3.9852	0.16233	4.0464	0.1255
745	4.015	0.15476	3.9688	0.16532	4.0261	0.12153
750	4.0067	0.15455	3.9592	0.1583	4.0097	0.11965
755	3.979	0.14984	3.9319	0.15873	3.9921	0.11762
760	3.9687	0.149	3.926	0.15605	3.9749	0.11452
765	3.9546	0.14577	3.9118	0.15454	3.9617	0.11422
770	3.9386	0.14733	3.899	0.14716	3.9498	0.11075
775	3.9217	0.1442	3.8898	0.15178	3.9342	0.10927
780	3.9136	0.13708	3.8721	0.14377	3.9212	0.10985
785	3.9127	0.14229	3.8594	0.14723	3.9133	0.10929
790	3.8948	0.14163	3.8463	0.14252	3.9023	0.10914
795	3.8853	0.14457	3.8369	0.14951	3.8884	0.10814
800	3.8607	0.1339	3.8281	0.14275	3.8839	0.10661
805	3.8493	0.14862	3.8242	0.14704	3.8699	0.10802
810	3.8543	0.13833	3.8152	0.14599	3.8588	0.105
815	3.8419	0.13944	3.8103	0.14411	3.8526	0.10372
820	3.8366	0.1377	3.7904	0.14833	3.8446	0.10301
825	3.8218	0.13384	3.7966	0.13969	3.8375	0.10308
830	3.8188	0.13272	3.7942	0.14769	3.8305	0.10294
835	3.8049	0.1355	3.7772	0.14329	3.8229	0.10139
840	3.7957	0.13606	3.777	0.13432	3.8134	0.10132
845	3.7814	0.14103	3.7651	0.1462	3.8065	0.10206
850	3.7983	0.13817	3.7593	0.14139	3.8024	0.10217
855	3.7873	0.12976	3.7534	0.1437	3.7923	0.097761
860	3.7721	0.12802	3.7489	0.14498	3.7865	0.098688
865	3.7609	0.13967	3.7459	0.13894	3.7829	0.10117
870	3.7525	0.13064	3.7407	0.14129	3.7779	0.10089
875	3.7504	0.13277	3.733	0.13208	3.7697	0.098302
880	3.749	0.13245	3.7339	0.13308	3.7645	0.098093
885	3.7494	0.12771	3.7245	0.13506	3.7597	0.09857
890	3.7507	0.12503	3.7133	0.12863	3.7546	0.098106
895	3.7348	0.12769	3.7121	0.13275	3.7498	0.097763
900	3.7305	0.13342	3.7048	0.13633	3.7443	0.098424
905	3.7231	0.13129	3.7045	0.13451	3.7398	0.097871
910	3.7173	0.13197	3.7065	0.13914	3.7359	0.097249
915	3.7126	0.13251	3.6967	0.13766	3.7313	0.097509
920	3.7056	0.13923	3.6897	0.13257	3.727	0.097305
925	3.7127	0.13301	3.6875	0.13715	3.7229	0.098209
930	3.7068	0.12879	3.6928	0.13281	3.7178	0.097795
935	3.6978	0.12756	3.6761	0.1361	3.7143	0.099327
940	3.6934	0.13052	3.6799	0.12969	3.7109	0.097141
945	3.6914	0.12977	3.6765	0.12749	3.7078	0.09625
950	3.6886	0.13782	3.6737	0.12456	3.7051	0.098084
955	3.6894	0.13452	3.6708	0.13453	3.6999	0.097999

960	3.6857	0.1339	3.66	0.12888	3.6974	0.097411
965	3.6795	0.12964	3.6607	0.13504	3.6908	0.097385
970	3.6736	0.13581	3.6509	0.13255	3.6874	0.096856
975	3.683	0.13931	3.6644	0.13727	3.6842	0.096808
980	3.6674	0.1338	3.6545	0.13241	3.6788	0.097124
985	3.6602	0.13559	3.6325	0.1357	3.6796	0.099659
990	3.6631	0.13305	3.6507	0.13053	3.6755	0.098649
995	3.656	0.13371	3.6474	0.13157	3.6729	0.099293
1000	3.6531	0.13736	3.6341	0.13545	3.6696	0.099894

	**7L**		**8L**		**9L**	
wavelength(nm)	n	k	n	k	n	k
290	3.4972	1.2998	3.6923	1.1447	3.7718	1.0328
295	3.5023	1.367	3.7013	1.178	3.7427	1.1995
300	3.4694	1.394	3.5908	1.3551	3.6599	1.2548
305	3.4036	1.4045	3.551	1.4391	3.6555	1.3201
310	3.336	1.5027	3.5225	1.3941	3.5823	1.3459
315	3.322	1.4274	3.4269	1.4865	3.5416	1.3057
320	3.27	1.479	3.4116	1.5259	3.4668	1.4714
325	3.2837	1.4556	3.4011	1.385	3.4815	1.3729
330	3.2944	1.3648	3.4731	1.3326	3.4809	1.4419
335	3.2633	1.3718	3.3561	1.4464	3.5402	1.3025
340	3.2637	1.3718	3.3961	1.4011	3.494	1.3792
345	3.2641	1.3386	3.4303	1.3006	3.4961	1.2528
350	3.3023	1.3121	3.4685	1.2757	3.5294	1.223
355	3.3719	1.299	3.5294	1.2627	3.5985	1.2029
360	3.4528	1.3035	3.6136	1.2677	3.6826	1.1982
365	3.5462	1.3336	3.709	1.2846	3.7724	1.2182
370	3.6376	1.3899	3.8074	1.3412	3.8714	1.2563
375	3.7289	1.4511	3.9068	1.3996	3.9637	1.3177
380	3.8197	1.5418	4.0057	1.4824	4.0666	1.3915
385	3.8902	1.6507	4.0967	1.5794	4.1601	1.5025
390	3.9566	1.7793	4.1635	1.7226	4.2379	1.6201
395	3.988	1.9267	4.2226	1.8519	4.294	1.7524
400	4.0118	2.062	4.2658	1.989	4.3405	1.8796
405	4.0171	2.1822	4.283	2.1322	4.3609	2.0221
410	4.015	2.2942	4.2965	2.2563	4.3832	2.1277
415	4.0147	2.3961	4.3059	2.3725	4.3728	2.275
420	4.01	2.4927	4.316	2.4728	4.3648	2.4102
425	4.0238	2.5792	4.3299	2.5766	4.4053	2.4629
430	4.0706	2.6375	4.357	2.6694	4.4043	2.5983
435	4.1378	2.6946	4.4244	2.7221	4.444	2.6994
440	4.2652	2.6905	4.5255	2.743	4.5095	2.768

445	4.4052	2.663	4.6486	2.7324	4.5844	2.8411
450	4.5663	2.581	4.7981	2.6589	4.7501	2.7706
455	4.7063	2.4605	4.9346	2.5516	4.8882	2.6874
460	4.8215	2.315	5.0494	2.4198	5.0155	2.563
465	4.9044	2.1545	5.1369	2.2637	5.1158	2.4116
470	4.9532	1.9918	5.1916	2.1083	5.1808	2.2625
475	4.9761	1.8365	5.2233	1.9509	5.2231	2.0897
480	4.9771	1.6824	5.2282	1.7873	5.2387	1.9169
485	4.9572	1.5402	5.2146	1.6371	5.2336	1.7691
490	4.9225	1.4136	5.1826	1.5037	5.206	1.6275
495	4.878	1.3075	5.141	1.3902	5.1648	1.5082
500	4.8251	1.2151	5.0897	1.2925	5.1132	1.3959
505	4.7719	1.1405	5.0342	1.2078	5.0632	1.3087
510	4.7172	1.0777	4.978	1.1377	5.002	1.2349
515	4.6645	1.0258	4.9221	1.0764	4.9488	1.1719
520	4.6139	0.98249	4.8702	1.0307	4.8958	1.1221
525	4.5651	0.94689	4.8203	0.98996	4.8426	1.0836
530	4.522	0.91761	4.771	0.95779	4.7954	1.0463
535	4.4782	0.89218	4.7243	0.93056	4.7499	1.0169
540	4.4403	0.87335	4.6828	0.9099	4.707	0.98963
545	4.4041	0.85673	4.645	0.89235	4.6695	0.9703
550	4.3703	0.84602	4.6095	0.87971	4.6325	0.95709
555	4.3375	0.83775	4.576	0.87198	4.6	0.94654
560	4.3095	0.8319	4.5456	0.8651	4.5676	0.93708
565	4.283	0.83031	4.5171	0.8632	4.541	0.93583
570	4.2577	0.83351	4.4911	0.86544	4.5113	0.93621
575	4.2347	0.84044	4.4695	0.87351	4.4871	0.9397
580	4.2144	0.85344	4.4499	0.88519	4.4684	0.95332
585	4.197	0.87124	4.4335	0.90199	4.4462	0.96934
590	4.1902	0.89627	4.424	0.92556	4.4355	0.99475
595	4.1932	0.92731	4.4263	0.95421	4.4391	1.0275
600	4.2113	0.96158	4.4429	0.98682	4.4556	1.0629
605	4.2497	0.99109	4.4772	1.0156	4.4912	1.0969
610	4.3046	1.0072	4.5292	1.0338	4.5487	1.1162
615	4.3719	0.99878	4.5922	1.0317	4.6197	1.1127
620	4.4325	0.96306	4.6579	1.002	4.688	1.0805
625	4.4818	0.90513	4.7114	0.9479	4.7432	1.0185
630	4.502	0.8389	4.74	0.88053	4.7696	0.9466
635	4.4977	0.77679	4.7446	0.81452	4.7704	0.87832
640	4.4752	0.73226	4.7273	0.76155	4.7502	0.82588
645	4.4491	0.70943	4.7029	0.72954	4.7219	0.79517
650	4.432	0.70719	4.6851	0.7185	4.702	0.79334
655	4.435	0.72109	4.6814	0.72287	4.699	0.80448
660	4.467	0.73742	4.704	0.73177	4.729	0.82186

665	4.5298	0.73315	4.7528	0.72707	4.7919	0.82357
670	4.6078	0.68726	4.8171	0.68928	4.8726	0.78365
675	4.666	0.59454	4.8704	0.61454	4.9433	0.69605
680	4.6761	0.48363	4.8903	0.51437	4.963	0.5781
685	4.647	0.38463	4.8732	0.41919	4.943	0.46674
690	4.598	0.3088	4.8323	0.33856	4.8923	0.38264
695	4.5437	0.25494	4.7814	0.2786	4.8365	0.31781
700	4.4906	0.21878	4.7296	0.23671	4.7787	0.27401
705	4.442	0.19383	4.6795	0.20579	4.7266	0.24176
710	4.3986	0.1754	4.6358	0.18508	4.6804	0.21788
715	4.3601	0.16272	4.5942	0.1686	4.6386	0.20133
720	4.3252	0.15274	4.5574	0.15747	4.5989	0.19057
725	4.2952	0.14619	4.5273	0.14864	4.5628	0.18154
730	4.2678	0.1395	4.4969	0.14141	4.5366	0.1743
735	4.2421	0.13593	4.4708	0.13618	4.5081	0.16678
740	4.217	0.13169	4.4458	0.13096	4.481	0.16298
745	4.1952	0.12704	4.4218	0.12653	4.4563	0.15832
750	4.1743	0.12604	4.4002	0.12364	4.4345	0.15283
755	4.1555	0.12129	4.3803	0.12012	4.4136	0.15051
760	4.1389	0.1201	4.3617	0.1169	4.3922	0.14691
765	4.1227	0.11758	4.3437	0.11588	4.3773	0.14221
770	4.1064	0.11639	4.3282	0.11193	4.3573	0.14208
775	4.0927	0.11393	4.3129	0.11032	4.3438	0.13904
780	4.0796	0.1128	4.2978	0.11047	4.3291	0.1358
785	4.0668	0.11275	4.2846	0.1064	4.3149	0.13477
790	4.0534	0.11062	4.2707	0.10698	4.3012	0.13296
795	4.0397	0.11007	4.2593	0.10523	4.286	0.12998
800	4.0301	0.10719	4.2458	0.10259	4.2751	0.12882
805	4.0179	0.10728	4.2331	0.10131	4.2621	0.12699
810	4.0077	0.10608	4.2227	0.099294	4.2491	0.12501
815	3.9976	0.10568	4.2115	0.097373	4.2399	0.12492
820	3.9891	0.10431	4.2017	0.096565	4.2289	0.12063
825	3.9809	0.10323	4.1931	0.095439	4.22	0.12048
830	3.9721	0.10319	4.1842	0.095125	4.2102	0.11856
835	3.9637	0.10154	4.1749	0.093883	4.1994	0.11879
840	3.955	0.10175	4.1656	0.093628	4.1892	0.11732
845	3.9489	0.10215	4.1581	0.092538	4.1822	0.11646
850	3.9379	0.09965	4.15	0.091605	4.1743	0.11646
855	3.9318	0.10056	4.1397	0.091592	4.164	0.11486
860	3.9243	0.09773	4.1329	0.091321	4.1572	0.1142
865	3.9194	0.097773	4.1253	0.088939	4.1489	0.11333
870	3.9123	0.096894	4.118	0.089321	4.1426	0.11253
875	3.906	0.096658	4.1108	0.086885	4.1345	0.11163
880	3.8986	0.095618	4.1026	0.086427	4.1266	0.10953

885	3.892	0.09492	4.097	0.086398	4.1205	0.10868
890	3.8872	0.095464	4.0899	0.086771	4.1144	0.10922
895	3.8807	0.093879	4.0835	0.08629	4.1077	0.1072
900	3.8756	0.093993	4.0769	0.085472	4.1013	0.10737
905	3.8676	0.092612	4.0717	0.085391	4.0954	0.10671
910	3.8641	0.094722	4.0659	0.085023	4.0873	0.10872
915	3.8589	0.092782	4.0599	0.085014	4.0827	0.10507
920	3.8547	0.093257	4.056	0.084889	4.0774	0.10645
925	3.85	0.09313	4.0507	0.084378	4.0728	0.10621
930	3.8441	0.092887	4.0461	0.085745	4.0668	0.10531
935	3.8401	0.091613	4.0421	0.084569	4.0631	0.10413
940	3.8355	0.092333	4.0365	0.083004	4.0568	0.10517
945	3.8314	0.092179	4.0302	0.084009	4.0512	0.10484
950	3.826	0.090759	4.0249	0.082722	4.0484	0.10433
955	3.8218	0.09071	4.0196	0.082297	4.0429	0.10295
960	3.8184	0.09035	4.0163	0.083586	4.0382	0.10119
965	3.8119	0.090305	4.0132	0.082068	4.034	0.10269
970	3.8114	0.089903	4.0075	0.08227	4.0285	0.10053
975	3.8056	0.091299	4.003	0.082816	4.0233	0.1022
980	3.8006	0.091263	3.9987	0.082014	4.0206	0.10192
985	3.798	0.090927	3.9955	0.083107	4.0154	0.10174
990	3.7938	0.091766	3.9911	0.08252	4.0111	0.10077
995	3.7913	0.091764	3.9878	0.082941	4.0073	0.1008
1000	3.7867	0.093497	3.9844	0.082239	4.0022	0.10074

10L

wavelength(nm)	n	k
275	3.8889	0.46559
280	3.9301	0.83355
285	3.8389	0.86449
290	3.8264	1.0317
295	3.7683	1.0961
300	3.7541	1.2344
305	3.7127	1.2669
310	3.6691	1.252
315	3.6259	1.275
320	3.5782	1.345
325	3.5906	1.2531
330	3.5811	1.2913
335	3.5242	1.3573
340	3.5243	1.3895

345	3.5694	1.2122
350	3.6053	1.1739
355	3.6695	1.1487
360	3.7566	1.148
365	3.8485	1.157
370	3.9498	1.2021
375	4.0538	1.2517
380	4.1583	1.3426
385	4.2525	1.4359
390	4.3427	1.5527
395	4.4126	1.6849
400	4.4668	1.8126
405	4.4984	1.9556
410	4.5164	2.0879
415	4.5229	2.2223
420	4.5349	2.3319
425	4.5421	2.4486
430	4.5628	2.5578
435	4.6021	2.6651
440	4.6889	2.7158
445	4.7219	2.8865
450	4.8609	2.8627
455	5.0147	2.798
460	5.1515	2.6832
465	5.2829	2.4814
470	5.3587	2.2918
475	5.4033	2.1126
480	5.4136	1.9448
485	5.4078	1.7579
490	5.3769	1.6211
495	5.33	1.4952
500	5.2755	1.377
505	5.2115	1.2789
510	5.1501	1.2042
515	5.0907	1.1464
520	5.032	1.0924
525	4.976	1.0438
530	4.9239	1.0179
535	4.8733	0.98939
540	4.8288	0.95548
545	4.7877	0.94788
550	4.7497	0.93046
555	4.7173	0.91882
560	4.6814	0.91307

565	4.6495	0.90966
570	4.6224	0.90747
575	4.593	0.91041
580	4.5699	0.92088
585	4.549	0.94077
590	4.5356	0.97007
595	4.536	1.0036
600	4.5524	1.0479
605	4.59	1.0794
610	4.6502	1.1032
615	4.7286	1.1016
620	4.8042	1.0707
625	4.8661	1.0051
630	4.8941	0.9276
635	4.8926	0.84516
640	4.8683	0.78793
645	4.8297	0.75324
650	4.8004	0.75122
655	4.7931	0.77326
660	4.8199	0.80189
665	4.8913	0.81809
670	4.9919	0.78371
675	5.0777	0.68249
680	5.1046	0.55076
685	5.0776	0.42479
690	5.0175	0.32756
695	4.9536	0.26415
700	4.891	0.21591
705	4.837	0.18612
710	4.7828	0.16645
715	4.7347	0.14724
720	4.6958	0.13861
725	4.6612	0.12378
730	4.6303	0.12181
735	4.5992	0.11637
740	4.5678	0.11279
745	4.5464	0.1092
750	4.5231	0.10622
755	4.4982	0.10033
760	4.4776	0.10159
765	4.46	0.095861
770	4.4429	0.096327
775	4.4271	0.092949
780	4.4096	0.092671

785	4.3944	0.0904
790	4.3814	0.089364
795	4.3657	0.089596
800	4.3543	0.085195
805	4.3403	0.086865
810	4.3274	0.08522
815	4.3174	0.081597
820	4.3074	0.080824
825	4.2978	0.081814
830	4.2874	0.080366
835	4.2757	0.077703
840	4.2655	0.078495
845	4.2572	0.081157
850	4.2466	0.077832
855	4.2395	0.079486
860	4.2321	0.078655
865	4.2248	0.075373
870	4.2151	0.07729
875	4.2097	0.074161
880	4.2004	0.071378
885	4.1926	0.072436
890	4.1867	0.073229
895	4.1784	0.071606
900	4.1725	0.074564
905	4.1657	0.07173
910	4.158	0.070905
915	4.1541	0.07066
920	4.1469	0.071455
925	4.1423	0.070668
930	4.1391	0.070459
935	4.1314	0.071227
940	4.1272	0.070284
945	4.1227	0.070336
950	4.117	0.068629
955	4.1106	0.068798
960	4.1089	0.070415
965	4.1046	0.069971
970	4.0976	0.070051
975	4.0931	0.068335
980	4.0894	0.067586
985	4.0849	0.068757
990	4.0802	0.067641
995	4.0755	0.067518
1000	4.0732	0.068066

References

S1 Yu, Y. F. *et al.* Controlled Scalable Synthesis of Uniform, High-Quality Monolayer and Few-layer MoS2 Films. *Sci Rep-Uk* **3**, 1866 (2013).

S2 Kresse, G. & Furthmuller, J. Efficient iterative schemes for ab initio total-energy calculations using a plane-wave basis set. *Physical Review B* **54**, 11169-11186, (1996).

S3 Becke, A. D. Density-Functional Exchange-Energy Approximation with Correct Asymptotic-Behavior. *Phys Rev A* **38**, 3098-3100, (1988).

S4 Shi, H. L., Pan, H., Zhang, Y. W. & Yakobson, B. I. Quasiparticle band structures and optical properties of strained monolayer MoS2 and WS2. *Physical Review B* **87**, 155304 (2013).
